# Supplementary material for: Diethanolamine Modified Perovskite-Substrate Interface for Realizing Efficient ESL-Free PSCs
Source: Nanomaterials (Basel). 2023 Jan 6;13(2):250. doi: 10.3390/nano13020250 (PMC9865489; doi:10.3390/nano13020250)
Supplement: Supplementary file 1 [file nanomaterials-13-00250-s001.zip › nanomaterials-2115052-supplementary.pdf]

nanomaterials

Supplementary Materials

Diethanolamine Modified Perovskite-Substrate Interface for Realizing Efficient ESL-Free PSCs

Sajid Sajid [1,2], Salem Alzahmi [1,2,*], Dong Wei [3], Imen Ben Salem [4], Jongee Park [5] and Ihab M. Obaidat [2,6,*]

1 Department of Chemical & Petroleum Engineering, United Arab Emirates University, Al Ain P.O. Box 15551, United Arab Emirates
2 National Water and Energy Center, United Arab Emirates University, Al Ain P.O. Box 15551, United Arab Emirates
3 College of Physics and Energy, Fujian Normal University, Fuzhou 350007, China
4 College of Natural and Health Sciences, Zayed University, Abu Dhabi P.O. Box 144534, United Arab Emirates
5 Department of Metallurgical and Materials Engineering, Atilim University, Ankara 06836, Turkey
6 Department of Physics, United Arab Emirates University, Al Ain P.O. Box 15551, United Arab Emirates
* Correspondence: s.alzahmi@uaeu.ac.ae (S.A.); iobaidat@uaeu.ac.ae (I.M.O.)

Supporting Information

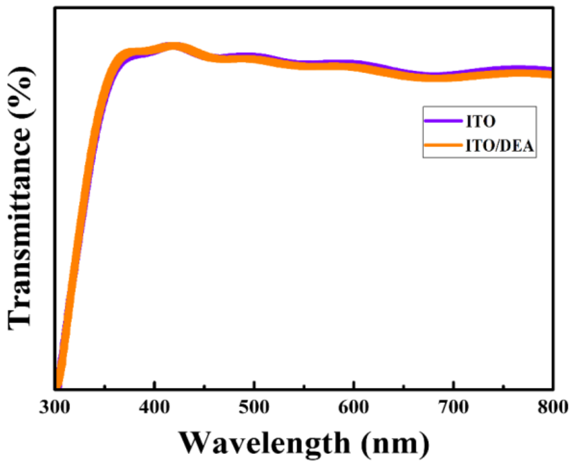

Figure S1. Transmittance spectra of bare ITO and DEA-treated ITO substrates.

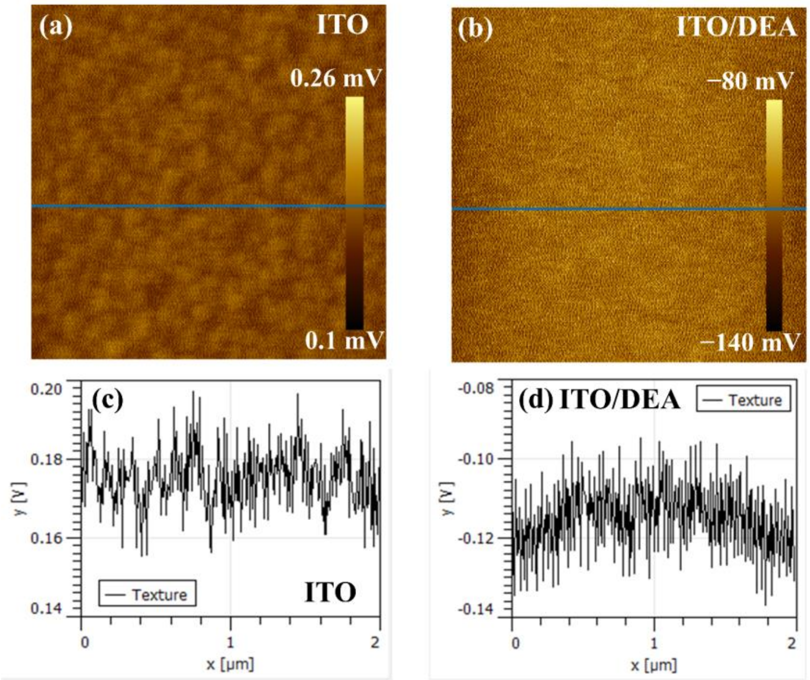

Figure S2. KPFM images (**a**,**b**) and corresponding potential difference curves (**c**,**d**) of the bare ITO and DEA-modified ITO substrates.

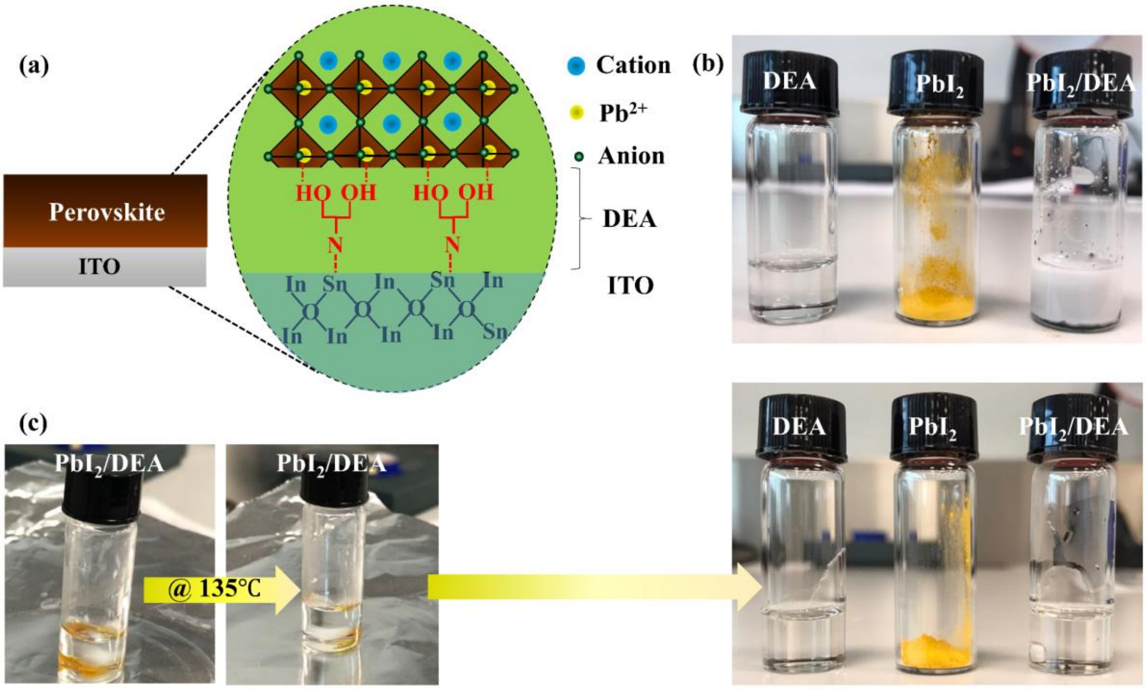

Figure S3. An strengthened link at the ITO/perovskite interface is highlighted by a coupling between the –OH group and Pb in the perovskite in this schematic example of surface modification of ITO-substrate with DEA (**a**), Photos of DEA liquid, PbI_2 powder and PbI_2/DEA mixture (**b**), Photos of DEA liquid, PbI_2 powder and PbI_2/DEA mixture under 135 °C (**c**).

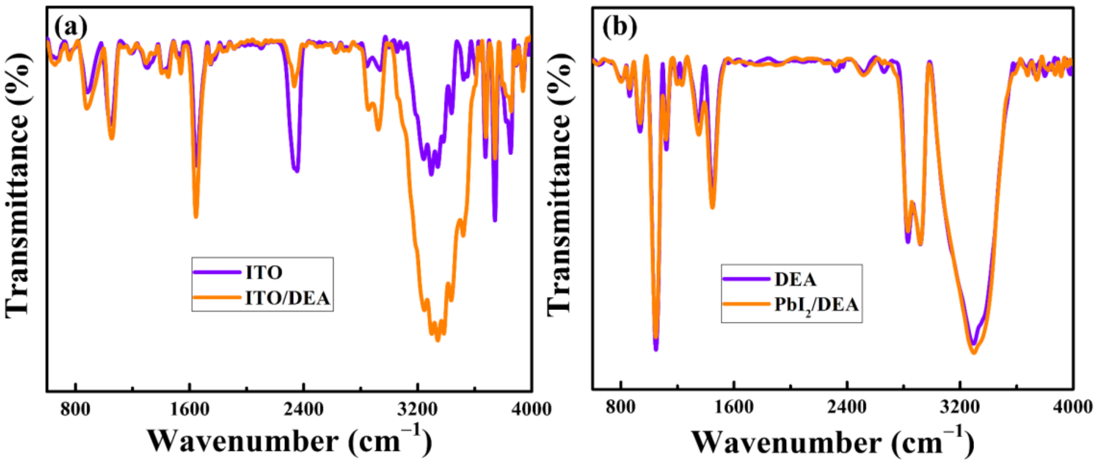

Figure S4. FT-IR spectroscopy of the ITO and ITO/DEA substrates (**a**), and DEA liquid and PbI_2/DEA mixture (**b**).

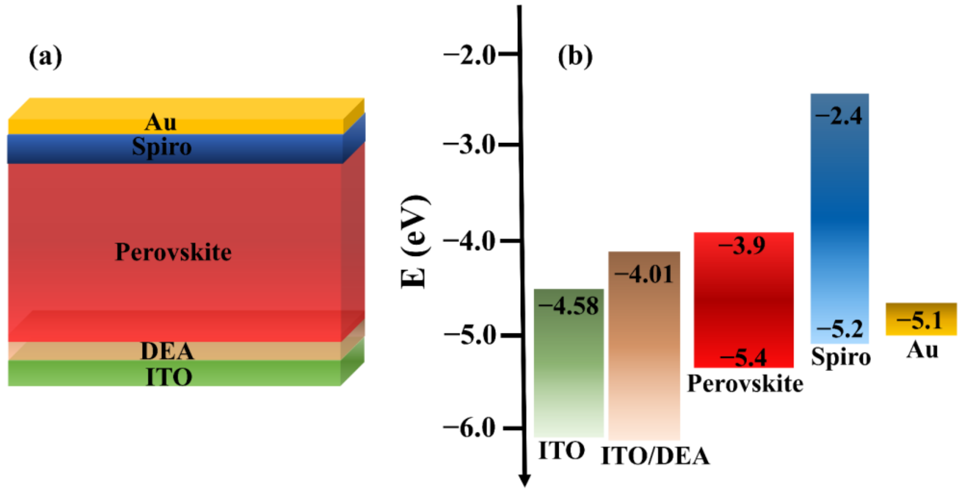

Figure S5. Schematic illustration of as-prepared PSC (**a**) and energy band diagrams of the corresponding layers (**b**).

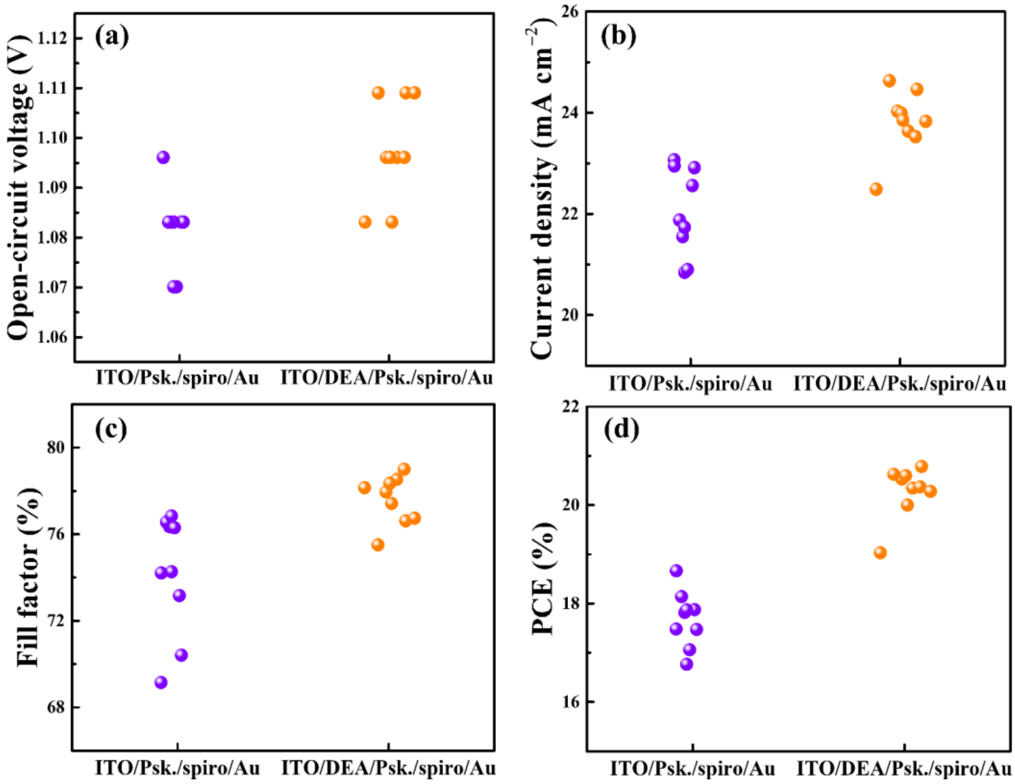

Figure S6. Statistical analyses of the ESLs-free PSCs prepared with bare ITO and DEA-modified ITO substrates, V_{oc} (**a**), J_{sc} (**b**), FF (**c**), and PCE (**d**).

Table S1. TRPL data for the perovskite layers prepared on bare ITO and DEA-modified substrates.

**Perovskite on**	**τ_1 (ns)**	**τ_2 (ns)**
ITO	10.95783 ± 0.195	111.30219 ± 1.58806
ITO/DEA	6.82892 ± 0.11677	48.19467 ± 0.39644
